# Impact of hyperglycaemia on cellular microenvironment and function of endometrium and uterine tube: scoping review focused on infertility in diabetic women

**DOI:** 10.3389/fcell.2025.1582039

**Published:** 2025-05-23

**Authors:** Peter Jackuliak, Martin Jankovský, Magdaléna Kovářová, Jaroslav Voller, Claudia Feitscherová, Ivan Varga

**Affiliations:** ^1^ Fifth Department of Internal Medicine, Faculty of Medicine, Comenius University and University Hospital Bratislava, Bratislava, Slovakia; ^2^ Faculty of Healthcare Studies, University of Western Bohemia, Pilsen, Czechia; ^3^ Institute of Histology and Embryology, Faculty of Medicine, Comenius University, Bratislava, Slovakia

**Keywords:** diabetes mellitus, hyperglycaemia, infertility, endometrial receptivity, embryo-endometrium crosstalk, endometrial immune cell, tubal infertility, hypothyroidism

## Abstract

**Introduction:**

Diabetes mellitus (DM) and associated comorbidities correspond to female infertility by many interrelated mechanisms. Yet most prior research focuses only on ovary dysfunction. Our work evaluates literature mechanisms of DM-induced uterine tube and endometrial dysfunction, corresponding impacts on female fertility, and potential evidence-based intervention targets.

**Methods:**

We conducted a scoping review (mapping review) follows the Joanna Briggs Institute (Manual for Evidence Synthesis, 2020 version). After identifying the research questions, we conducted a comprehensive search across four electronic databases by entering the keyword “diabetes”, with a combination with other keywords as the uterus, endometrium, uterine/Fallopian tube, infertility and embryo implantation. We excluded manuscripts that address the issue of gestational diabetes. Most of these studies were in animals.

**Results:**

There is compelling evidence for connecting DM with uterine tube infertility *via* endometriosis, thyroid dysfunction, and susceptibility to infectious disease. DM damages the endometrium before pregnancy *via* glucose toxicity, lesions, excessive immune activity, and other mechanisms. DM also hinders endometrium receptivity and embryo–endometrium crosstalk, such as through disrupted endometrium glucose homeostasis. We also hypothesize how DM may affect the function of immune cells in uterine tube and uterus, including changes in the number and types of cells of innate and acquired immunity, disrupting immunological barrier in uterine tube, alterations in formation of neutrophil extracellular traps or polarization of macrophages.

**Discussion:**

We discuss evidence for clinical practice in terms of glycaemic control, lifestyle modifications, and medical interventions. For example, there is currently substantial evidence from rodent models for using metformin for increase in endometrial thickness, number of stromal cells and blood vessels and restoration of normal endometrial architecture, and bariatric surgery for recruitment of protective immune cell types to the endometrium. We also briefly highlight the future prospects of stem cells, artificial intelligence, and other new approaches for managing DM-associated female infertility. Further studies are necessary for optimizing female reproductive outcomes.

## 1 Introduction

Diabetes mellitus (DM) is a common and chronic metabolic disease, characterised by hyperglycaemia secondary to absolute/relative insulin deficiency and/or insulin resistance due to pancreatic Langerhans islet beta-cell dysfunction, often accompanied by features of metabolic syndrome (American Diabetes Association Professional Practice Committee, 2022). Approximately 537 million adults (20–79 years) globally are living with diabetes; the vast majority exhibit type 2 diabetes mellitus (T2DM). The total number of people living with diabetes is projected to increase to 643 million by 2030 and 783 million by 2045. These projections indicate that one in eight adults will be living with diabetes in 2045 (Ogurtsova et al., 2017; Cho et al., 2018). But over the past few years, the frequency of less common type–the type 1 diabetes mellitus (T1DM) - worldwide has also increased, from 2% to 5% (Alzhanuly and Sharipov, 2024). People living with diabetes have an increased risk of developing complications. The most common complications affect the heart, blood vessels, eyes, kidneys, nerves, teeth, gums and association with carcinogenesis (Kupcova et al., 2023; Addanki and Sumathi, 2024; Mirestean et al., 2023). Yet diabetes also impacts a rarely discussed aspect of health: reproductive health. DM may lead to disruption of normal sexual and reproductive function in men and women *via* diabetic-induced end organ damage and psychological stress. Whilst the incidence of sexual problems increases with age, these problems are also present in young adults, mostly those with type 1 diabetes mellitus (Jacobson et al., 2015).

Infertility is defined as a failure to achieve pregnancy within 12 months of unprotected intercourse or therapeutic donor insemination in women younger than 35 years, or within 6 months in women older than 35 years; and affects up to 15% of couples (Infertility Workup for the Women’s Health Specialist, 2019). Large numbers of people are affected by infertility in their lifetime. Approximately 17.5% of the adult population—roughly 1 in 6 worldwide—experience infertility; indicating the urgent need to increase access to affordable, high-quality fertility care for those in need (Cox et al., 2022; Njagi et al., 2023).

On 4 April 2024, the National Vital Statistics System of the Centres for Disease Control and Prevention released the final “Births” report for 2022 as well as the provisional “Births” report for 2023. Both reports attested to a worrisome trend for maintaining current population levels: a decline in the general fertility rate among women ages 15–44 and thus in the attendant annual number of live births in the United States (Adashi et al., 2024). The reasons for the decline in fertility include health, social, and societal factors. To a lesser but substantial extent, the increase in patients with diabetes and who are of reproductive age also contributes to the decline in fertility. An increased number of male patients with DM have been reported in childbearing age and the DM prevalence is closely associated with the decline of fertility (Lutz, 2006). Diabetes can impact male fertility in many ways; such as erectile dysfunction, ejaculatory dysfunction (either retrograde ejaculation or a complete lack of ejaculation), or testicular dysfunction—including reduced testosterone synthesis, decreased spermatogenesis, increased germ cell apoptosis, and semen abnormalities (Badejogbin et al., 2024; Graziani et al., 2024). The most commonly discussed problem in diabetes is erectile dysfunction in men with diabetes. The pathophysiology of erectile dysfunction in DM consists of vascular, hormonal, and neurologic insults (Gandhi et al., 2017). Diabetic neuropathy may impair autonomic and somatic nerve processes essential for erections. Diabetes is also associated with impaired relaxation of cavernosal smooth muscle due to endothelial-derived nitric oxide induced by glycosylation products (Patel et al., 2017). Men with diabetes may also be at increased risk of low serum testosterone levels, which may lead to a decline in sexual desire and directly or indirectly to erectile dysfunction (Lockie et al., 2024; Grossmann et al., 2008). Various experimental and clinical studies reveal that DM is associated with worse conventional sperm parameters, reaching particularly low values, compared with the general population. T1DM can influence the expression of genes involved in sperm DNA repair; resulting in a high rate of nuclear DNA fragmentation, mitochondrial DNA deletions with mitochondrial respiratory chain alteration, and subsequent decreased sperm motility (Condorelli et al., 2018). Recent studies in animal models suggest that mesenchymal stem cells may be the future of improving diabetes-induced male reproductive dysfunction and semen parameters (Lu et al., 2024; Kocamaz et al., 2025).

A less-discussed problem is reproductive dysfunction in women with diabetes. They often experience lower rates of fertility than women who do not have diabetes. There are multiple factors associated with diabetes that can make it difficult for women to achieve a pregnancy; such as obesity, being underweight, having diabetic complications, having polycystic ovary syndrome (PCOS), or having an autoimmune disease. These conditions can lead to irregular or absent periods, premature menopause, or higher risk for endometrial cancer. The prevalence of PCOS in women with T1DM is higher than in the general population (Łebkowska et al., 2024). PCOS is the most common endocrinopathy affecting reproductive-aged women, with impacts across the lifespan from adolescence to post-menopause. PCOS affects 10%–13% of women of reproductive age and has many causes. The PCOS Society revised the diagnostic criteria for PCOS (Teede et al., 2023) to when all of the following criteria apply: (1) ovarian dysfunction (oligo-ovulation or polycystic ovaries on an ultrasound scan), (2) clinical or biochemical hyperandrogenism, and (3) other related disorders associated with hyperandrogenism are excluded (e.g., Cushing’s syndrome). PCOS occurs when there is peripheral insulin insensitivity and subsequent hyperinsulinaemia, which together with elevated luteinising hormone act on ovarian theca cells and lead to increased androgen production. Elevated androgen levels prevent normal follicular maturation, causing infertility. Hyperinsulinemia often leads to T2DM and metabolic syndrome, increasing the risk of cardiovascular diseases (Azziz et al., 2009), and depressive and anxiety symptoms (Çetintulum Aydın et al., 2025).

In general, diabetic females have problems such as delayed menarche, irregular menstrual cycle, subfertility, complications in pregnancy, and early menopause. Diabetic females also have the negative effect of oxidative stress on the reproductive system (Andlib et al., 2024). Most corresponding scientific articles evaluate only dysfunction of the ovary in diabetes. Our scoping review reveals the mechanisms of diabetes-induced tubal and endometrial dysfunction, corresponding impacts on fertility, and potential intervention targets.

## 2 Methods

This scoping (mapping) review follows the Joanna Briggs Institute Manual for Evidence Synthesis (Peters et al., 2024). Firstly, we identified the research questions: impact of diabetes on the cellular microenvironment of uterine tube and uterus and the process of implantation and embryo-endometrium crosstalk. As a next step, we conducted a comprehensive search across four electronic databases (Web of Science, Scopus, Google Scholar, and PubMed/MEDLINE) by entering the keyword “diabetes”, with a combination with other keywords as the uterus, endometrium, uterine/Fallopian tube, infertility and embryo implantation. Articles in English with full-text or with an explanatory abstract were included into the study. Then, articles were assessed by two experts with more than 20 years of clinical and research experiences, one endocrinologist/diabetologist and one expert in reproductive medicine and clinical embryology, for relevance to the subject (selecting the evidence). We excluded all manuscripts that address the issue of gestational diabetes, which has a different pathomechanism of development and only appears during pregnancy (we focused on infertility). A data/evidence extraction and analysis tool were used to systematically collect data from the included studies, followed by a narrative synthesis to summarize and interpret the findings. We choose this approach due we would like to explore the breadth of the literature, map and summarize the evidence (from basic to clinical research), identify knowledge gaps and inform future research directions (Munn et al., 2018). Additionally, this scoping review can be a precursor to a further systematic review.

Although the use of artificial intelligence (AI) methods in writing review articles is on the rise, we did not use any of the options offered by current modern technology. The use of AI tools in writing scoping reviews has some critical limitations. Current AI tools can struggle with factual accuracy, citation errors, and a lack of deep contextual analysis (Thurzo and Varga, 2025).

## 3 Diabetes and tubal infertility

Uterine tubes are responsible for cardinal processes needed for successful reproduction; including the uptake and transportation of oocytes, transport of spermatozoa, fertilization, and transport of the fertilized ova and early-stage embryo towards the uterine cavity. The interaction of the tubal epithelium with the spermatozoa facilitates sperm functions, selection, and activation (capacitation). Moreover, the uterine tube also provides a particular microenvironment that *in vivo* is crucial for early embryo nutrition and development (Csöbönyeiová et al., 2022; Varga et al., 2022). Diabetes is a negative factor for women’s reproductive health, especially in relation to endometrial pathologies and impaired embryo implantation. There is less knowledge about the negative impact of diabetes on the reproductive function of the uterine tubes. Nevertheless, diabetes is an independent risk factor of tubal infertility (Egbe et al., 2020).

**FIGURE 1:**
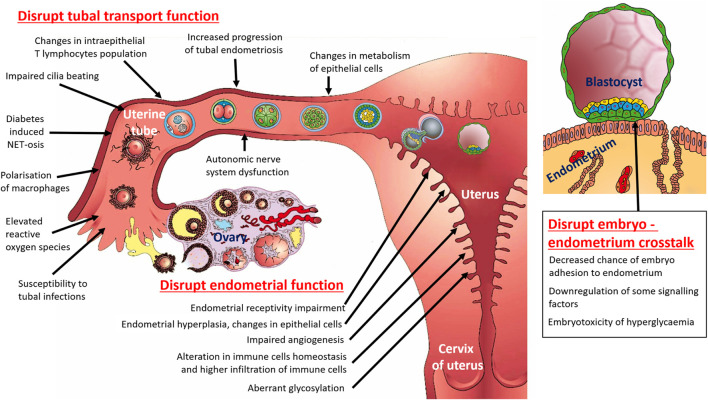
Schematic diagram summarizing the multifactorial pathways by which diabetes affect female fertility (with focus on uterine tube, uterus and embryo – endometrium crosstalk).

The association between diabetes and tubal infertility (Figure 1) is an area of growing interest in medical research. Whilst the direct link between diabetes and tubal infertility is not fully understood, several mechanisms and factors may contribute to this association. It is interesting that the opposite association—tubal infertility/tubal blockage—is associated with increased risk of both T2DM (Tobias et al., 2015) and gestational diabetes (Tobias et al., 2013). However, it is possible that tubal infertility is diagnosed earlier than diabetes itself, which indicates that reproductive health is a sensitive barometer of overall health.

### 3.1 Diabetes and disrupt tubal transport function

Diabetes is associated with tubal infertility due to its systemic multifactorial effects on vascular, autonomic nervous, immune, and hormonal systems; which impact the transport function of the uterine tubes. Diabetes can probably disrupt the transport function of ciliated epithelium lining the uterine tubes. This damage may result from chronic hyperglycaemia leading to oxidative stress: elevated reactive oxygen species levels may impair the frequency of cilia beating and reduce the effectiveness of oocyte/early embryo transport (Kaltsas et al., 2023). However, the cited authors do not support this conclusion with any experimental results. In the scientific literature, we found only an indirect connection between increased oxidative stress (even in general, not especially caused by diabetes) and tubal transport disruption. In women with ectopic tubal pregnancy (as a result of disrupted tubal transport), it is possible to detect a higher level of oxidative stress (Tok et al., 2021; Üreyen Özdemir et al., 2022). Although ectopic tubal pregnancy can also be a consequence of a previous inflammation of the uterine tube, it may not be only in connection with impaired ciliary function due to higher oxidative stress. Therefore, we consider this connection between diabetes and impaired ciliary beating to be speculative for now.

Diabetes has been linked to an increased progression of endometriosis (Alhallak et al., 2023); which can involve the uterine tubes and lead to scarring, blockages, or adhesions. However, this link is only partially true *vice versa*: endometriosis is not a risk marker for T2DM (Vaduva et al., 2023), but endometriosis increases the risk of gestational diabetes (Salmeri et al., 2023). Tubal endometriosis significantly impacts the transport mechanism of the female oocyte, male spermatozoa, and early-stage embryo inside the uterine tube (Xia et al., 2018; Nassir et al., 2024). *In vitro*, the peritoneal fluid from women with endometriosis decreases the tubal ciliary beat frequency. This impairment of ciliary action in women with endometriosis might reduce fertility (Lyons et al., 2002). Therefore, it is also possible that endometriosis without direct anatomical effects on the uterine tubes may negatively affect tubal transport function.

Hypothyroidism–as a common DM associated thyroid dysfunction - affects the size of tubal epithelial cells in rabbits (presence of larger-sized ciliated cells in the entire uterine tube, and larger-sized secretory cells in the isthmus of uterine tube), probably also changes the metabolism of these cells, and may impact tubal reproductive function (Anaya-Hernández et al., 2015).

### 3.2 Diabetes and tubal immune cells

In general, clinical and subclinical inflammation in the female reproductive organs significantly reduces fertility - that’s why it is important to think about how diabetes can affect innate and acquired immune cells in the uterine tube. The uterine tube is an immunologically privileged organ and should tolerate allogenic sperm and semi-allogenic embryos without eliciting an inflammatory immune response. The immune cells–mostly intraepithelial regulatory T lymphocytes of the uterine tube probably represent a type of “immunological barrier” (Visnyaiová et al., 2024; Varga et al., 2019).

The role of tubal neutrophils is largely unknown. From veterinary reproductive medicine studies is known, insemination always stimulates neutrophil migration into the female reproductive tract, which eliminates excess spermatozoa and bacterial contaminants introduced by the breeding process (Alghamdi and Foster, 2005). In 2004, a new mechanism of how polymorphonuclear cells such as neutrophils clear away microbes was described–the formation and release of web-like DNA structures and antimicrobial proteins called neutrophil extracellular traps (NETs) (Brinkmann et al., 2004; Janko et al., 2023; Tonello et al., 2025). In general, the human spermatozoon is a sufficient stimulus to trigger the release of NETs. Neutrophils trap spermatozoa by enmeshing them through NETs. This direct cell contacts between the neutrophils and spermatozoa, which can result in the entrapment of sperm cell, represents the first stage of sperm phagocytosis by neutrophils (Zambrano et al., 2016). NETs formation should be considered in future studies of reproductive failure, as these extracellular fibres and NET-derived pro-inflammatory capacities will impede proper oocyte fertilization (Rivera-Concha et al., 2023; León et al., 2024). In relation to diabetes, recently more studies show that NETs perform as double-edged sword. On one side, NETs could repress the infection-related inflammation; on the other side, excessive production of NETs may have severe impact on the organic damage and be involved in many inflammatory diseases, such as T1DM, T2DM, and diabetes-induced complications (Zhu et al., 2023). However, in relation to diabetes-induced tubal infertility and the formation of NETs, ​​we still need to answer three emerging questions.

1) Does such NETs formation inside the uterine tube also occur *in vivo*? All the above-cited experiments with NETs formation in co-culture of neutrophils and male spermatozoa were performed with neutrophils isolated from peripheral blood. But it is known that, uterine tube neutrophils exhibit a phenotype distinct from peripheral blood neutrophils (Smith et al., 2006).
2) Tubal epithelial cells produce and release to tubal fluid prostaglandin 2 after luteinizing hormone stimulation which suppress the phagocytic activity of neutrophils for sperm *in vivo* (Marey et al., 2013).
3) There is no experimental or clinical data on how diabetes affects the formation of NETs inside the uterine tube and whether it contributes to increased sperm capture before fertilization and subsequent infertility - it is only a hypothesis derived from the fact that diabetes affects the formation of NETs in other organs.

Macrophages are also essential immune cells critical to normal reproductive functions, exhibiting significant adaptability that allows for the transformation into various phenotypes in response to their surrounding environment. Macrophages exhibit functional plasticity. M1 macrophages serve as essential component of the immune system’s response to infections, characterized by their potent pro-inflammatory properties. Following an inflammatory response, an anti-inflammatory response is required to restore immune homeostasis. This is marked by a phenotypic transition wherein M1 macrophages polarize toward the M2 phenotype. The primary functions of M2 macrophages include the repair and remodelling of damaged tissues, participation in angiogenesis and secretion of anti-inflammatory cytokines (Ghamangiz et al., 2025; Chen et al., 2023). In uterine tubes, macrophages are localized within the epithelium and lamina propria and exhibit cyclic changes in numbers during menstrual cycle (Gaytán et al., 2007). The normal function of uterine tube macrophages can be diverse. During sperm phagocytosis, macrophages form extracellular traps as a possible mechanism of sperm selection within the uterine tubes (Lu et al., 2023). Several studies have confirmed that the number of macrophages significantly increases in ectopic pregnancies when compared to normal uterine tubes (Shaw et al., 2011; Wang et al., 2020). The role of macrophages in the pathogenesis of tubal ectopic pregnancy is not clear, but macrophages might dysregulate both tubal motility and smooth muscle contraction of the uterine tube (Visnyaiová et al., 2024). In tubal inflammatory conditions such as salpingitis and hydrosalpinx, an increase in the number of macrophages has been reported. In particular, M1 macrophages, producing proinflammatory cytokines (IL-6 and IL-8), are predominant (Yoshino and Ono, 2025). In case of diabetes, persistent hyperglycaemia and oxidative stress have been shown to synergistically exacerbate the polarization propensity of M1 macrophages, leading to sustained secretion of potent pro-inflammatory mediators. In diabetic patients with wound healing problems (as a common clinical complication of diabetes), the diabetic microenvironment stimulates migration of monocytes to the wound and they transform into M1 macrophages. This exacerbates the inflammatory response at the wound site and impedes normal wound healing. Therefore, the persistence of the pro-inflammatory M1 phenotype and deficiency of M2-type macrophages in diabetic wounds may contribute to an unbridled pro-inflammatory microenvironment (Song et al., 2025). There are no experimental or clinical data on how diabetes and a hyperglycaemic microenvironment affect the function and polarization of macrophages in the uterine tube. We can only assume that diabetes also causes a switch of macrophages to M1 more easily in the diabetic uterine tube, which will damage the transport function of the uterine tube (causing infertility and ectopic pregnancy).

Tubal intraepithelial immune cells are mostly intraepithelial regulatory T-lymphocytes (Varga et al., 2019). Hypothyroidism–which is often associated with diabetes - can influence immune cells in the uterine tube by increasing the number of intraepithelial lymphocytes in the ampulla, whilst decreasing in the isthmus (Méndez-Tepepa et al., 2020). It is therefore possible that hypothyroidism damages the immunological barrier between the lumen of the uterine tube (with sperm) and the wall of the uterine tube, thereby contributing to tubal infertility.

Additionally, DM is hypothesized to increase susceptibility to infectious diseases (Benfield et al., 2007), including increased frequency and severity of urogenital infections (Fünfstück et al., 2012); e.g., pelvic inflammatory disease, *chlamydia*, or gonorrhoea. These infections can cause tubal scarring, blockages, or hydrosalpinx—all of which contribute to tubal infertility.

### 3.3 Diabetes and tubal autonomic nerve dysfunction

Diabetic autonomic neuropathy, autonomic nerve system dysfunction, is a serious and common complication of diabetes. Diabetic autonomic neuropathy—along with vasculopathy; connective tissue damage; and other endocrine, nutritional, and pharmacological factors—may influence reproductive functions (Verrotti et al., 2014) and hypothetically can reduce tubal smooth muscle contractility and peristalsis. Estimating the significance of diabetic autonomic neuropathy in relation to disrupting tubal transport function is speculative, as there is no direct evidence from experimental publications. However, diabetic autonomic neuropathy negatively affects many pelvic organs and is associated with various complications, including urinary bladder and sexual dysfunction (Agochukwu-Mmonu et al., 2020).

## 4 Impacts of diabetes on the endometrium before pregnancy

Untreated or improperly treated diabetes affects the morphology and function of the endometrium (uterine mucosa, Figure 1). Hyperglycaemia facilitates endometrial hyperplasia, as a precancerous condition of endometrial carcinoma (Zhou et al., 2020); and causes abnormal uterine bleeding (Vygivska et al., 2024) and endometrial carcinoma (Wang et al., 2022). In women diagnosed with endometrial hyperplasia, DM is a risk factor for coexistent cancer, and thus may be included in a predictive algorithm for risk stratification (Raffone et al., 2020). Precise etiological links between diabetes and endometrial pathologies are mostly unknown. The underlying biological mechanisms involved in endometrial pathologies in diabetic patients—including hyperglycaemia, insulin resistance, hyperinsulinemia, changes in epithelial-to-mesenchymal transition, chronic inflammation, and obesity—may contribute to an increased risk of endometrial carcinoma in diabetic patients (Wang et al., 2022). The prevalence of hypothyroidism in diabetic patients is higher than in the general population, particularly in those with T1DM due to the shared autoimmune nature of both conditions; hypothyroidism is another mechanism that facilitates uterine hyperplasia (Rodríguez-Castelán et al., 2019).

### 4.1 Diabetes and morphological changes of endometrium

Chronic exposure to a glucose-rich environment creates several physiological and pathophysiological changes. In experimental diabetes-induced rats, histological examination of the endometrium indicates tissue oedema, changes in the morphology of the uterine glands, the presence of inflammatory cells, and a decrease of CD45 positive (so-called leukocyte common antigen) cells (Nacar et al., 2016). A hyperglycaemic cellular microenvironment mediates irreversible cell damage or changes in cell proliferation. Glucose is toxic when high levels deleteriously affect cells and tissues (Giri et al., 2018). There are several pathways, such as glycosylation, by which hyperglycaemia induces toxicity. Glycosylation is one of the most common protein post-translational modification events, in which diabetic patients with increased plasma glucose levels exhibit proportionally more glycation. Aberrant glycosylation can influence multiple cellular properties; including cell signalling, proliferation, transformation, differentiation, apoptosis, migration, and invasion (Sharma et al., 2024). N-acetylgalactosaminyltransferase 2 (GALNT2) enzyme can modify the epidermal growth factor receptor glycosylation and activity, and thereby may enhancing cell proliferation within the endometrium of diabetic patients (Zhou et al., 2020). Another mechanism may be increased expression of neuronal and endothelial nitric oxide synthase (nNOS and eNOS, respectively) in the diabetic uterus (Karabulut and Sonmez, 2021); which may affect cell communication, immune reactions, and vascular functions. However, there are more possible mechanisms by which chronic hyperglycaemia can lead to endometrial changes, such as by impaired angiogenesis and endothelial dysfunction. Yet disrupted angiogenesis has until now been described only during placental development in the case of gestational diabetes and not in the endometrium before pregnancy (Zhou et al., 2016; Huang et al., 2024; Milan et al., 2024). Additionally, endometrial carcinoma cell culture experiments indicate that high glucose inhibited cell apoptosis, facilitated cell cycle progression, and enhanced the adhesion and invasion activity of endometrial cancer cells by mediating the upregulation of Snail and downregulation of E-cadherin expression (Han C. et al., 2015).

Diabetes has the potential to facilitate the progression not only of anatomically normally localised endometrium in the uterine cavity, but also of endometriosis lesions (Alhallak et al., 2023). Results of the mentioned immunohistochemical study confirmed changes in steroid hormone receptor levels inside endometriosis lesions (in stromal and epithelial compartments), increased macrophage abundance (immune activation is associated with endometriosis progression), and reduction of phosphate and tensin homolog (PTEN) expression (PTEN is essential for maintaining cellular homeostasis by regulating cell proliferation, survival, and metabolism).

### 4.2 Diabetes and endometrial immune cells

The innate and adaptive immune mechanisms are key components of regulation of reproductive function of uterus and its endometrium. In recent years, views on the importance and functioning of the endometrial immune cells during blastocyst implantation, placentation, and subsequent pregnancy have changed significantly. During early pregnancy, uterine natural killer (NK) cells are the most abundant cell type at the maternal–embryonal interface, comprising 70% of the total lymphocytes in the endometrium in the third month of pregnancy before undergoing a decline (Fu and Wei, 2021; Lapides et al., 2023). Approximately 20%–30% of women with idiopathic recurrent miscarriages or recurrent implantation failure exhibit altered uterine NK cell counts (Kuon et al., 2017; Lapides et al., 2022). In pregnant mice, experimentally induced hyperglycaemia alters immune homeostasis, including NK cell proportion and function in peripheral blood and endometrium (Xiong et al., 2024).

Endometrial macrophages are likely to play an essential role during the menstrual cycle, especially in the menstrual context of tissue degradation, which requires regulated repair, regeneration, and phagocytic clearance of endometrial tissue debris to re-establish tissue integrity in preparation for pregnancy as they have a role in angiogenesis and wound healing in other tissues (Ma et al., 2022). Macrophages play an important role in the development of endometriosis lesions and the concomitant inflammation. Nowadays, endometriosis has been referred to as ‘a disease of the macrophages’, as macrophages are abundant in lesions where they are recruited and undergo alternative activation. Macrophages also play a role in enhancing inflammation, following with neutrophil recruitment through the release of chemokines (Abramiuk et al., 2022). M2 macrophages predominate in lesions and are involved in collagen fibres formation (fibrogenesis) in endometriosis lesions (Duan et al., 2018). Since diabetes contributes to the development of endometriosis, a chronic disease in women that also causes infertility, it is possible that one of the mechanisms is through the increasing the number and changing in polarisation of macrophages.

Obesity is also often associated with diabetes. Obesity itself causes M1 macrophage numbers to increase (although typically this occurs in adipose tissue, accompanied by adipose tissue inflammation and insulin resistance). On the other hand, anti-inflammatory M2 macrophages are typical in the adipose tissue of slender individuals (Castoldi et al., 2016; Chylikova et al., 2018). Metformin, a drug used to treat hyperglycaemia, can influence the polarization of macrophages toward M1 and M2 phenotypes. The ability of metformin to support M2 polarization and suppress M1 polarization could enhance its anti-inflammatory properties and potentiate its protective effects in conditions such as chronic inflammatory diseases (Jafarzadeh et al., 2025). There are already initial indications that metformin and a higher presence of M2 macrophages could be helpful in placentation and improving pre-eclampsia in pregnancy (Shen et al., 2025).

In contrast to macrophages and NK cells recruited to the pregnant uterus from the first weeks, neutrophils are barely found until the second trimester where a novel pro-angiogenic decidual neutrophil population has been identified (Amsalem et al., 2014). Changes in endometrial neutrophil behaviour and neutrophil extracellular traps formation have so far only been described in the case of gestational diabetes. Neutrophil activity is indeed altered in gestational DM, exhibiting pronounced activation and spontaneous generation of NETs by isolated neutrophils in *in vitro* culture (Stoikou et al., 2017). Also, Shen et al. (2021) demonstrated that hypoadiponectinemia in gestational DM is the cause of NETs formation and NETs promoting trophoblast apoptosis. It is hypothetically possible that an excessive neutrophil activity in gestational DM could contribute to the development of preeclampsia (Vokalova et al., 2018). It is therefore pertinent to ask whether pre-gestational diabetes also changes the activity of neutrophils in the endometrium and thus, for example, reduces endometrial receptivity and the possibility of implantation? In other hand, embryonic trophoblast cells preventing neutrophil activation and inhibiting NETs formation through vasoactive intestinal peptide-mediated pathways (Calo et al., 2017). Diabetes facilitates the progression of endometriosis and endometriosis elevates peripheral blood NETs content (Sun et al., 2025). However, a direct link between endometrial NETosis and diabetes has not been described so far.

For the sake of completeness, we add that hypothyroidism (which is often associated with diabetes) also causes endometrial hyperplasia and higher infiltration of immune cells into the endometrium. Since most experimental animal models only evaluate the isolated effect of diabetes or hypothyroidism on endometrial tissue, it is still unclear in clinical practice which of these two factors has a greater impact on uterine pathology or whether it is a combined effect (Rodríguez-Castelán et al., 2019).

## 5 Impacts of diabetes on endometrial receptivity and embryo–endometrium crosstalk

Endometrial receptivity, a key determinant of pregnancy success, refers to the ability of the endometrium to support embryo implantation. Inadequate endometrial receptivity often results in embryo implantation failure and miscarriage (Lessey and Young, 2019; Liu et al., 2024). Endometrial epithelium—a simple columnar epithelium composed of ciliated cells and secretory cells—plays a critical role in the initial stages of embryo implantation because it provides the first physical contact site for the blastocyst. Endometrial receptivity is complicated and can be regulated by various signalling pathways. During the narrow period during the hormonally regulated menstrual cycle when the endometrium is optimally receptive to the implantation of an early-stage embryo, the “implantation window,” epithelial cells undergo structural and biochemical changes. Secretory epithelial cells form specific projections from the plasma membrane termed pinopodes and produce membrane-associated glycoprotein mucin 1 (Wu et al., 2019). Endometrial receptivity is often disrupted in diabetic patients due to a combination of cellular, metabolic, hormonal, immune, and vascular abnormalities. Receptive endometrium is the first step of embryo implantation (Ashary et al., 2018). Implantation and subsequent decidualisation of the endometrial stroma during early-stage pregnancy depend on proper embryo–endometrium crosstalk. This synchronized dialogue includes an intricate interplay among epithelial, immune, and stromal cells; hormones; immune factors; cytokines; exosomes; and adhesion molecules—underpinning the process of implantation, placenta formation, and further embryo development (Tan et al., 2024). Unfortunately, this stage of development is commonly termed a “black box” because of its inaccessibility, as it occurs inside the uterus. Nowadays, with tissue engineering methods and various cultivation systems, it is possible to form endometrial two-dimensional models to three-dimensional organoids and novel assembloids that can recapitulate many aspects of endometrial tissue architecture and cell composition during implantation (Kleinová et al., 2024; Kim et al., 2025).

### 5.1 Diabetes and endometrial receptivity impairment

Implantation failure or miscarriage is an important reason for infertility in diabetic women. This implantation failure depends upon the degree of metabolic control of diabetes in the first trimester (Greene, 1999). Causes of endometrial receptivity impairment and disrupt embryo–endometrium crosstalk in diabetic women may be.

• *Decreased chance of early-stage embryo adhesion to the endometrial epithelium:* Diabetes increases integrin gene expression in the endometrium at the time of embryo implantation, which can lead to disorganized cell-to-cell or cell-to-extracellular matrix adhesion (Bakhteyari et al., 2019).
• *Altered endometrial epithelial cells functional morphology:* Disrupts differentiation/transformation of surface epithelial cells before and during implantation due to changes in the expression of cytoskeletal proteins and their modifying enzymes, contributing to changes in cell shape (Keller et al., 2024) and regulating posttranslational modification of proteins involved in the maintenance of epithelial cell polarity (Ruane et al., 2024). Cytoplasmic projections of secretory epithelial cells—pinopodes, ultrastructural markers of receptive endometrium—are reduced and poorly developed in diabetic mice (Albaghdadi and Kan, 2012; Ma et al., 2021), and membrane-associated glycoprotein mucin one is overexpressed in endometrial epithelial cells (Albaghdadi and Kan, 2012).
• *Downregulation of some signalling factors; including cytokines, growth factors, and homeobox transcription factors important for endometrial receptivity and placentation:* Expression of insulin-like growth factor 1 (IGF-1), leukaemia inhibitory factor (LIF), and Beclin-1 (a key protein involved in autophagy) is decreased in endometrial epithelial cells; and IGF-1 expression is decreased also in decidual cells in diabetic women (tissue obtained after miscarriages) (Gurbuz et al., 2019). Among the mentioned signalling factors, LIF plays a pivotal role in implantation, is abundantly expressed in the glandular epithelium during the implantation window phase, and is induced in the stroma surrounding attached blastocysts (Aikawa et al., 2024). Decreased expression of LIF was also described in endometrial epithelial cells of diabetic mice (Albaghdadi and Kan, 2012; Ma et al., 2021). Zhou et al. (2021) described that less-differentiated epithelium for implantation in mice is present in the case of absence of epithelial IGF-1 receptors, and additionally epithelial IGF-1 receptors are activated by IGF-1 produced in endometrial stromal cells (this production is also decreased in diabetic women).
• *Maternal hyperinsulinemia:* In insulin-treated mice a significant increase of endometrial phosphorylated mechanistic target of rapamycin (p-mTOR) is present (Li et al., 2017). The mTOR pathway is an important negative regulator of autophagy that plays a positive role in early pregnancy by positively regulating decidualisation and trophoblast invasion; as well as regulates the infiltration, enrichment, and functional regulation of decidual immune cells (Li et al., 2024).
• *Disrupted endometrial glucose homeostasis:* Over-activation of the important regulator of glycogen metabolism, the adenosine monophosphate-activated protein kinase (AMPK) in an animal model of T1DM (Zhang et al., 2020), but not in T2DM (Ma et al., 2021).
• *Immune dysregulation creating an endometrial microenvironment less conducive to implantation:* Albaghdadi and Kan (2012) described overexpression of interferon gamma (IFNG) in the uterus of diabetic mice; probably associated with nonreceptive endometrium and embryo loss. IFNG secreted during pregnancy by uterine NK cells acts as a negative regulator of trophoblast invasion (Verma et al., 2018).
• *Vascular defects:* In diabetes, endometrium during pregnancy (so-called decidua) has morphologically detectable vascular changes that likely contribute to embryo loss and birth defects. Burke et al. (2007) described in diabetic mice impaired endometrial spiral artery modification, including fewer spiral arteries in the implantation site and a smaller lumen diameter of spiral arteries. This results in an abnormal blood supply to the endometrium, leading to possible structural and functional defects during placentation and embryo development.

### 5.2 Diabetes and disrupt embryo–endometrium crosstalk

However, disruption of the synchronized molecular and cellular dialogue between the endometrium and the embryo in the case of maternal diabetes may not only occur due to impaired function of the endometrial tissues. There are also possible causes in the context of the embryo, because of which embryo–endometrium crosstalk may be disrupted (Figure 1).

• *Embryotoxicity of glucose:* In *in vitro* cell cultures, hyperglycaemic conditions are toxic to early-stage embryos (Sutton-McDowall et al., 2006). Also, *in vivo* animal studies support the findings that impaired pre-implantation embryo development, and increases DNA damage and protein O-GlcNAcylation (Brown et al., 2018), can potentially disrupt embryo–endometrium crosstalk.
• *Changes in stress-related receptors of the blastocyst:* Seeling et al. (2018) described in rabbits higher expression of alfa-2A adrenergic receptors in trophoblasts cells than in embryoblast cells, whilst in normoinsulinemic blastocysts this expression was reversed (higher in embryoblast cells). The function of these receptors in this early stage of embryonal development is unknown.

Thus, it is evident that diabetes can significantly alter complex communication between the embryo and endometrium during and after implantation through metabolic, hormonal, immune, and structural changes. This disruption can negatively affect implantation, pregnancy establishment, and maintenance.

## 6 Potential intervention targets

**FIGURE 2:**
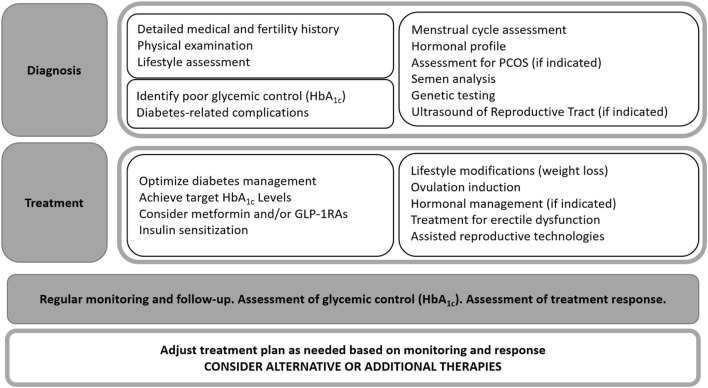
Algorithm outlining the treatment approach for fertility disorders.

In clinical practice there are general recommendations to improve embryo–endometrium crosstalk in diabetic women (Figure 2).

• Glycaemic control: Tight glucose control before and during pregnancy is crucial to minimise adverse effects on the endometrium and embryo.
• Lifestyle modifications: Weight management, a healthy diet, and regular exercise can improve insulin sensitivity and overall reproductive health.
• Medical interventions (Table 1): Medications such as metformin (for insulin resistance) or low-dose aspirin (to improve blood flow) may be beneficial in some cases. In recent years there has also been discussion about the effect of the new group of antidiabetic drugs termed glucagon-like peptide-1 receptor agonist (GLP-1RA).

**TABLE 1:** Summarizing of mechanisms of the three most common used antidiabetic drugs in treatment of fertility disorders of diabetic patients.

Antidiabetic drug	Effect in fertility
Metformin	**Improved ovulation and menstrual regularity** in women, mostly with PCOS and obesity. Metformin improves insulin sensitivity, which can help regulate hormone levels, leading to more regular menstrual cycles and improved ovulation. Some studies suggest that metformin, especially when combined with other fertility drugs like clomiphene citrate, can improve pregnancy rates in women with PCOS. **Reduced risk** of ovarian hyperstimulation syndrome in women with PCOS undergoing IVF treatment. Reduced risk of miscarriage in women with PCOS. Studies on metformin's impact on sperm parameters (count, motility, morphology) have yielded mixed results. Some studies show no significant effect on sperm count, while others suggest potential improvements in morphology and chromatin packaging. Conversely, some animal studies indicate a decrease in sperm count and motility with metformin use. There is some evidence suggesting metformin might have a beneficial effect by targeting the vascular system in erectile dysfunction. Studies have shown neutral or even beneficial effects on testosterone level.
Thiazolidinediones Glitazones	Improvement in insulin sensitivity in women with insulin resistance and PCOS but also in obese men. Glitazones can help restore ovulation in women with PCOS and improve ovulation. Decreased in androgen levels (such as testosterone), which is often elevated in women with PCOS, thus potentially improving menstrual regularity and fertility. Mixed results on live birth rates (some RCTs found little to no significant increase in live birth rates compared to other treatments like clomiphene citrate. In males, some studies have explored the impact of glitazones on semen quality (improvements in parameters like sperm concentration and motility). The weight loss associated with glitazone therapy could lead to improvements in fertility parameters.
GLP-1 receptor agonists (GLP-1RA)	Improved fertility through weight loss. This can manifest as more regular menstrual cycles, improved ovulation, and increased pregnancy rates. Weight loss achieved with GLP-1RAs in obese men may positively impact fertility by improving hormone profiles and semen quality. Direct stimulating role – potential anti-inflammatory and anti-fibrotic effects in the ovaries and endometrium, which could be beneficial for fertility. GLP-1RAs have shown promise in improving menstrual regularity and potentially increasing fertility rates in overweight and/or obese women with PCOS in the preconception period. Some studies even suggest they can reverse polycystic ovary morphology and decrease androgen levels. There are increasing reports of unplanned pregnancies in women using GLP-1RAs, potentially due to improved ovulation as they lose weight. Some small early studies and animal research suggest GLP-1RAs might have a beneficial impact on semen parameters like sperm concentration, motility, and morphology. They may also improve sperm metabolism and insulin secretion in vitro. Animal studies have shown mixed results regarding the effect of GLP-1RAs on testosterone levels, with some indicating a decrease and others showing no significant change.

Many studies indicate that metformin, when used to treat PCOS, significantly reduced serum androgen levels, improved insulin sensitivity, restored menstrual cyclicity, and was successful in triggering ovulation. As a result, metformin may be useful for treating PCOS-related infertility (Attia et al., 2023). Fewer studies have evaluated the direct effects of metformin on the endometrium. In animal models, orally administrated metformin led to an increase in endometrial thickness compared with sham endometrium. Metformin exhibited a significant increase in the number of endometrial glands, stromal cells, and blood vessels (Imran et al., 2024) and restores normal endometrial architecture (Carnovale et al., 2025). Also, other drugs such as clomifene citrate and thiazolidinediones (e.g., rosiglitazone and pioglitazone) are often used to increase insulin sensitivity and decrease insulin resistance; these drugs are considered to be first-line ovulation-inducing drugs in infertile women with PCOS either alone or in combination with metformin (El-Khayat et al., 2016).

GLP-1RA therapy has potential for reversing female infertility. Previously, it was thought that weight loss could correct hormonal imbalances and consequently restore ovulation. However, the effects of GLP-1RA on the endometrium—crucial for embryo implantation—remain unclear (Sola-Leyva et al., 2025). GLP1-RA treatment was associated with substantial improvement in homeostasis model assessments: insulin resistance, body mass index, waist circumference, sex hormone binding globulin level, and a slight reduction in total testosterone level compared with a control group. A decrease in total body fat was evident in European populations. GLP1-RA monotherapy was not superior to metformin in terms of free testosterone, dehydroepiandrosterone sulphate, and free androgen index (Zhou et al., 2023).

Lifestyle interventions aimed at improving fertility in women living with obesity accompanied by T2DM are imperative given the interconnected nature of these conditions. Balanced, minimally processed, plant-based diets—with low glycaemic load meals and moderate fat and fibre intake—hold promise in terms of supporting fertility among women with obesity and T2DM; more studies focusing on this population are necessary to comprehensively assess fertility outcomes through lifestyle modifications (Gitsi et al., 2024). However, if we consider that the most important factor that positively affects fertility is weight, bariatric surgery is also beneficial. Bariatric surgery can help improve fertility and pregnancy outcomes in several ways. Excess weight may trigger hormone imbalances that can affect a person’s ovulation cycles and impart difficulties to getting pregnant. Excess weight can also increase the chances for conditions that affect fertility. Bariatric surgery effectively increased levels of sex hormones (Moxthe et al., 2020). Obese females had a significant decrease in total and free testosterone after bariatric surgery. Bariatric surgery also led to lower E2 levels and increased luteinizing hormone, follicle-stimulating hormone, and sex hormone binding globulin levels. Sexual function reflected by Female Sexual Function Index scores also improved (Abdullah et al., 2022). Weight loss in women after bariatric surgery corresponded to significant reductions in serum CRP and IL-6, but not TNF-α levels. Tissue immune cell densities in endometrium were unchanged except for “protective” CD8^+^ lymphocytes, which increased significantly with weight loss and play important role in immune surveillance in endometrial cancer prevention. Tissue CD3^+^ lymphocytes density correlated negatively with systemic IL-6 levels (Naqvi et al., 2022).

### 6.1 Future possible or alternative therapies

The near future may bring three more new methods that can restore fertility in patients with diabetes. First, application of mesenchymal stem cells due to their regenerative effects and their participation in several paracrine pathways can improve the fertility outcome (Chatzianagnosti et al., 2024). According to Pala et al. (2014), in a rodent model, stem cells are not even needed directly, but growth factors as the granulocyte colony-stimulating factor affecting stem cells are sufficient for regeneration of the diabetes damaged endometrium. Second, artificial intelligence has the potential to improve infertility diagnosis and assisted reproduction techniques outcomes—with possible applications such as ultrasound monitoring of folliculogenesis, endometrial receptivity, embryo selection based on quality and viability, and prediction of post-implantation embryo development—in order to eliminate potential contributing risk factors (Medenica et al., 2022). Third, recent findings suggest a promising role of autologous platelet-rich plasma in enhancing endometrial cell differentiation, promoting vascular regeneration, and, most importantly, increasing endometrial thickness (Stefanović et al., 2025).

Fertility disorders are addressed by various fields of medicine. Many pathogenetic mechanisms are still poorly understood and therefore treatment is often problematic and insufficiently effective. Many results come from animal studies and are only indirectly applied to human medicine. Another problem is that results from animal models are not confirmed in human medicine. Many new treatment modalities are under discussion in terms of managing fertility disorders: use of stem cells, immunological treatment (in case of autoimmune aetiology), and many others. This article is also intended to contribute to the opening of new research questions and possibly new discussions regarding managing patients with DM.

## 7 Limitations

We identify two major limitations of present study. The first is that we cannot clearly confirm which endometrial and tubal pathology is caused only by diabetes and chronic hyperglycaemia, and which pathologies often associated with diabetes - such as hypothyroidism (Biondi et al., 2019), obesity, PCOS (Zhu et al., 2021), chronic inflammation, or presence of diabetes-associated autoantibodies - also play an important role. Especially, DM and thyroid dysfunction often coexist in patients. The close association between diabetes and hypothyroidism is primarily due to autoimmune mechanisms, metabolic interactions, and shared risk factors. The prevalence of hypothyroidism (including subclinical hypothyroidism) in individuals with T1DM varies in different countries and ethnic groups from 7% to 35% in both sexes (Medenica et al., 2024; Han J. et al., 2015; Talwalkar et al., 2019). It is still unclear in clinical practice which of these two factors–diabetes or hypothyroidism - has a greater impact on tubal and/or uterine pathology or whether it is a combined effect.

The second limitation is that most of studies describe the association of diabetes and uterine/tubal infertility in rodent models (mice, rats, rabbits), or mini-pigs and the use of different drugs limits the translational potential some of the described results. Knowledge about the normal function of immune cells in the reproductive tract is usually based on knowledge from veterinary embryology, breeding of cattle, dogs and horses. Experimental induction of diabetes in laboratory animals may include administration of low-dose streptozotocin, causing pancreatic beta-cell dysfunction; and/or feeding a high fat diet, causing insulin resistance (Gheibi et al., 2017). Additionally, genetically modified mice—such as non-obese diabetic mice or knockout models—are used to study the genetic and autoimmune aspects of diabetes (Pearson et al., 2016). Surgical removal of the pancreas can also be a feasible approach for advanced diabetes research (Heinke et al., 2016). Each method has its advantages and limitations, depending on the research goals. Based on current experimental (laboratory animal-based) research focused only on diabetes, it is impossible to predict unequivocally whether diabetes alone has an impact on fertility, whether the impact is more pronounced in the presence of diabetes-associated disorders, or whether diabetes-associated disorders have a more significant negative impact than hyperglycaemia itself. Moreover, fertility in rodent models and humans differs significantly in several biological and physiological aspects (poly-ovulatory cycles in rodents instead of mono-ovulatory cycles in humans, superficial implantation in rodents instead of deep trophoblastic invasion in humans, short reproductive cycle and large litter sizes in rodents, *etc.*) (Garretson et al., 2023; Biondic et al., 2023). Therefore, the results of many of the aforementioned animal experimental studies cannot be directly transferred to human clinical practice. Rodent models are invaluable for advancing basic reproductive biology and developing therapeutic approaches due to their rapid reproduction, genetic manipulability, and cost-effectiveness. However, due to critical differences in reproductive physiology, findings in rodents must be cautiously extrapolated to humans. Integrative approaches combining animal models, human tissue/organoid models, and clinical studies are essential for translational success in human reproductive medicine.

## 8 Conclusion

Thirty years ago, women with diabetes and possible infertility were recommended an individual approach within reproductive medicine centres (Briese and Müller, 1995). Since then, researchers have been trying to better understand the biological mechanisms that impair endometrial receptivity, embryo–endometrial crosstalk, or tubal transport function in diabetic women. Diabetes definitively contributes to sexual dysfunction and consequently to subfertility. Yet there are many open questions regarding the pathophysiology of the problems, which can be different in males and females. We mentioned the limitations of prior experimental studies. In clinical practice, more longitudinal clinical studies with larger sample sizes are necessary to better comprehend the connection between diabetes and sexual dysfunction and infertility, mainly in females. Understanding and dividing the role of fertility and sexual issues in reproductive dysfunction can help guide evaluation and management.

Diabetes can significantly alter embryo–endometrium crosstalk through metabolic, hormonal, immune, and structural changes. Proper management of diabetes and its associated conditions is essential to optimize endometrial receptivity and improve reproductive outcomes in diabetic women. Recent findings nevertheless point to the importance of screening patients with infertility for DM, and *vice versa* (Medenica et al., 2024). Women with pregestational diabetes are advised to plan their pregnancies to optimize glycemia and reduce fertility and pregnancy complications (Chimenea et al., 2024).

## Glossary

AI: artificial intelligence
AMPK: adenosine monophosphate-activated protein kinase
CRP: C-reactive protein
DM: diabetes mellitus
DNA: deoxyribonucleic acid
eNOS: endothelial nitric oxide synthase
GALNT2: N-acetylgalactosaminyltransferase two
GLP-1RA: glucagon-like peptide-1 receptor agonist
IFNG: interferon gamma
IGF-1: insulin-like growth factor 1
LIF: leukaemia inhibitory factor
mTOR: mechanistic target of rapamycin
NETs: neutrophil extracellular traps
NK cell: natural killer cell
nNOS: neuronal nitric oxide synthase
O-GlcNAcylation: O-linked-N-acetylglucosaminylation
PCOS: polycystic ovary syndrome
p-mTOR: phosphorylated mechanistic target of rapamycin
PTEN: phosphate and tensin homolog
T1DM: type 1 diabetes mellitus
T2DM: type 2 diabetes mellitus
TNF-α: tumour necrosis factor α
